# Social Support, Everyday Discrimination, and Depressive Symptoms Among Older African Americans: A Longitudinal Study

**DOI:** 10.1093/geroni/igaa032

**Published:** 2020-08-10

**Authors:** Weidi Qin, Ann W Nguyen, Dawne M Mouzon, Tyrone C Hamler, Fei Wang

**Affiliations:** 1 Jack, Joseph and Morton Mandel School of Applied Social Sciences, Case Western Reserve University, Cleveland, Ohio; 2 Edward J. Bloustein School of Planning and Public Policy, Rutgers, The State University of New Jersey, New Brunswick

**Keywords:** Black older adults, Family social support networks, Friendship social support networks, Mental health, Unfair treatment

## Abstract

**Background and Objectives:**

The purpose of the study was to explore the long-term effects of everyday discrimination on depressive symptoms among older African Americans, as well as the moderating role of social support in this association.

**Research Design and Methods:**

Mixed-effects negative binomial regression analyses were performed on data selected from 6 waves of the Health and Retirement Study (2006–2016; baseline *N* = 1,144). The number of depressive symptoms was calculated based on an 8-item Center for Epidemiologic Studies Depression measure. Everyday discrimination was measured using a 6-item scale. Contact with and perceived support from extended family and friends were assessed.

**Results:**

Older African Americans who experienced more frequent perceived discrimination had more depressive symptoms over time. Significant interactions between discrimination and perceived support from extended family and friends were found, indicating that among older African Americans who reported higher support from extended family and friends, perceived discrimination was positively associated with depressive symptoms over time. However, perceived discrimination and depressive symptoms were not longitudinally related among those who reported lower levels of perceived support.

**Discussion and Implications:**

This is one of the few studies to examine the effects of discrimination on depressive symptoms over time and the first longitudinal study to test the role of social support in coping with discrimination in older African Americans. This study extends cross-sectional works on discrimination and mental health, indicating that experiences of discrimination can result in worse mental health over time. The significant interactions are consistent with the resource mobilization framework, which suggests that individuals who are more negatively affected by discrimination (more depressive symptoms) are more likely to reach out to friends and family to cope with discrimination.

**Translational Significance**: Older African Americans who experience more discrimination and depressive symptoms are more likely to seek support from extended family and friends for coping with discrimination over time. The study findings suggest that older African Americans may benefit from interventions based on stress-coping resources via support from extended family and friends in coping with discrimination.

## Background and Objectives

### Background

Being African American and experiencing discrimination are inexorably connected in the United States. A recent report indicated that African Americans report extensive experiences of discrimination at institutional and individual levels (National Public Radio, Robert Wood Johnson Foundation & Harvard T.H. Chan School of Public Health, 2017). Discrimination has been linked to adverse psychological outcomes such as depression (Hudson et al., 2015; Mouzon et al., 2017) and places individuals at risk for overall health disparities. Older African American adults remain an underexamined population who are more likely to have experienced prolonged exposure to discrimination and, thus, are at higher risk of being negatively affected by its effects. The current cohort of older African American adults is a unique population, as they have experienced de jure discrimination during Jim Crow and the continued detrimental effects of overt and covert discrimination in multiple facets of their lives. These discriminatory laws presumably threatened not only their safety, but also their health and well-being (Nadimpalli et al., 2015). Moreover, over the course of their lives, older African Americans have experienced discrimination that occurs in social encounters and discrimination in the form of segregated neighborhoods, political disenfranchisement, under-resourced schools, and employment (Mouzon et al., 2017). From a life course perspective, the cumulative exposure to discrimination that older African Americans have faced uniquely contributes to their negative mental and physical health outcomes. The purpose of this study was to examine the association between discrimination and depression over time among older African Americans and the moderating effects of social support in this association.

### Discrimination and Depression

Discrimination is a form of unfair treatment that more often affects disadvantaged populations. Two broad classes of discrimination—major lifetime discrimination and everyday discrimination—are studied in the health literature (Kessler et al., 1999; Williams et al., 1997). Major lifetime discrimination refers to singular instances of unfair treatment in consequential life domains such as housing, the labor market, and the educational system. For example, lifetime discrimination encompasses experiences such as being prevented from buying, renting, or leasing a home; being unfairly fired or denied a promotion; or unfairly denied a bank loan. Everyday discrimination, on the other hand, refers to routine instances of typically more minor hassles such as receiving poorer service at restaurants and being followed around in stores. Major lifetime discrimination tends to tap into discrete or acute incidents of unfair treatment, whereas everyday discrimination has a more chronic and ongoing nature.

Exhaustive reviews of the literature have consistently linked both major and everyday discrimination to adverse mental health outcomes (Lewis et al., 2015; Paradies et al., 2015; Williams & Mohammed, 2013). For example, data from the National Survey of American Life (NSAL) show that blacks who reported high levels of everyday discrimination had higher odds of serious psychological distress than those who reported low levels (Chae et al., 2011). Despite that fewer studies focused on older adults, one study using data from the Health and Retirement Study (HRS) found that everyday discrimination was associated with more frequent depressive symptoms among older African Americans, who also reported higher rates of both lifetime and everyday discrimination than older whites and Hispanics (Ayalon & Gum, 2011). Everyday discrimination is also strongly associated with more frequent symptoms of psychological distress (Nguyen, Taylor et al., 2017) and depressive symptoms (Marshall-Fabien & Miller, 2014) among older adults in the NSAL. Some evidence suggests that among older adults, everyday discrimination, as a chronic stressor, is more strongly associated with mental health outcomes than major lifetime discrimination (Ayalon & Gum, 2011).

Few studies have examined the relationship between discrimination and depression longitudinally, especially among older adults. Longitudinal studies are important for determining the temporal ordering of discrimination and depression. That is, are individuals who perceive more frequent discrimination more likely to later develop depression, or are individuals who have depression more likely to perceive discrimination or people more likely to treat individuals presenting with observable symptoms of depression unfairly? Longitudinal evidence strongly suggests that it is in fact discriminatory experiences over time that lead to the onset of depression. Luo et al.’s (2011) investigation demonstrated this relationship in a national probability sample of older adults; they found that respondents who reported experiences of major discrimination in 2006 reported more depressive symptoms 2 years later than respondents who did not report experiences of major discrimination in 2006. Similarly, a longitudinal study of new immigrants in Hong Kong indicated that respondents who reported more experiences of discrimination at the beginning of the study had more depressive symptoms when they were reassessed 1 year later (Chou, 2012). Research on African Americans indicates a similar pattern. An investigation using data from the Baltimore area found that African American respondents who reported more frequent incidences of racial discrimination at Wave 1 reported more depressive symptoms at Wave 3 (English et al., 2014). Schulz et al.’s (2006) study of discrimination among African American women in the Detroit metropolitan area had similar findings, indicating that an increase in experiences of discrimination over time predicted an increase in depressive symptoms over time. Taken as a whole, these longitudinal studies suggest that experiences of discrimination can lead to the onset of depressive symptoms.

### Social Support and Mental Health Among African Americans

Research has shown that social support can protect against a range of mental health problems, including depression, anxiety, and psychological distress (Chatters et al., 2015, 2018; Nguyen et al., 2019). For example, studies on depression have found that African Americans who receive more frequent emotional support and are in more frequent contact with extended family members are less likely to meet the criteria for major depressive disorder (Lincoln & Chae, 2012; Taylor et al., 2015). Research on anxiety disorders has identified the beneficial effects of social support on posttraumatic stress disorder (PTSD) and social anxiety disorder (Levine et al., 2015; Nguyen, Chatters, Taylor, & Mouzon, 2016). In particular, studies in this area have indicated that emotional closeness to friends can protect against social anxiety disorder (Levine et al., 2015) and emotional support from family can protect against PTSD (Nguyen, Chatters, Taylor, Levine et al., 2016). Empirical evidence has also demonstrated that social support can protect against suicidal ideation and suicide attempts (Nguyen, Taylor et al., 2017).

While there is a paucity of research on the role of social support in the mental health of older African Americans, the few studies that have examined this association have identified the protective qualities of social support in this population. Among older African Americans, social support has been identified as a protective factor against depression, depressive symptoms, and serious psychological distress (Chatters et al., 2015). In a nationally representative sample of older African Americans, Chatters et al. (2015) found that respondents who reported receiving more frequent support from family and church members had fewer depressive symptoms and lower levels of serious psychological distress. In examining social support among cancer patients, Hamilton et al. (2013) found that respondents who reported receiving more emotional support from family members were less likely to be diagnosed with depression. Research on social support among older African Americans has also indicated that not only can it protect against mental illness, but it also can promote well-being. Walls’s (1992) study identified a positive association between support from family and church members and feelings of well-being. Furthermore, a more recent analysis of specific dimensions of subjective well-being among older African Americans indicated that social support from family and friends predicted higher levels of life satisfaction, happiness, and self-esteem (Nguyen, Chatters, Taylor, & Mouzon, 2016).

### Discrimination, Social Support, and Mental Health

In addition to promoting well-being and mental health, social support is a stress-coping resource that is particularly important for older African Americans (Nguyen, 2018). There is a growing body of evidence that points to the stress-buffering effects of social support. In particular, investigations in this area have indicated that social support can attenuate the negative effects of discrimination on mental health. For instance, in examining the role of social support from church members among older African Americans, Nguyen (2018) reported that frequency of contact with and emotional closeness to church members buffered against the effects of discrimination on generalized anxiety disorder (GAD). For respondents who had low levels of contact and emotional closeness, discrimination was associated with a greater likelihood of meeting the criteria for GAD. In contrast, for respondents who had high levels of contact and emotional closeness, discrimination and GAD were unrelated. Research examining the stress-buffering effects of social support among older women in South Korea also found that social support moderated the relationship between discrimination and depression (Lee & Kim, 2016). More specifically, the findings indicated that discrimination was linked to depression via stress. Social support reduced respondents’ stress levels and thus reduced the probability of depression.

Although a number of studies have suggested that social support can act as a stress buffer in the association between discrimination and mental health, at least one study has identified a different pattern. Nguyen, Taylor et al.’s (2017) analysis of discrimination among African American men found that among men in late adulthood, the association between discrimination and serious psychological distress was stronger among those who reported high levels of support than those who reported low levels of support. This pattern of findings indicated that resource mobilization may be operating in this sample. That is, respondents who experienced more acute psychological effects of discrimination may be reaching out to their social networks to mobilize the support that they need for coping with discrimination and the ensuing distress. Additionally, acutely distressed respondents’ network members may have recognized their visible signs of distress and mobilized around them to provide increased support. In reviewing the extant research, it is unclear what the role of social support is in the discrimination–mental health connection. Is social support a stress buffer or social resource that is mobilized in response to a stressor? It may be that social support functions in accordance with the stress-buffering model or the resource mobilization framework depending on the specific contexts and populations.

### Study Objectives

Existing research on discrimination, social support, and depressive symptoms among older African Americans has been largely restricted to cross-sectional designs or community-based samples. Little is known about whether social support moderates the adverse effects of discrimination on depressive symptoms in the long term among older African Americans at the population level. Given that cumulative experiences of everyday discrimination are a distinct stressor disproportionately experienced by older African Americans, a longitudinal examination of the moderating role of social support in the relationship between discrimination and depressive symptoms is important for several reasons. First, the present study on the longitudinal effects of social support would further our understanding of how social support from friends and extended family functions over time for older African Americans who are affected by everyday discrimination. Second, our study findings could inform health professionals working with older African Americans of potential support systems and help them identify possible coping resources that can provide long-term beneficial effects for mental health.

The present study aims to examine (a) the association between discrimination and depressive symptoms over time, (b) the association between social support and depressive symptoms over time, and (c) the moderating effect of social support in the relationship between discrimination and depressive symptoms over time in a nationally representative sample of older African Americans. The primary hypotheses for the present study are (a) increased discrimination is associated with more depressive symptoms over time, (b) receiving more frequent support is associated with fewer depressive symptoms over time, and (c) more frequent social support buffers the negative effects of discrimination on depressive symptoms over time.

## Research Design and Methods

### Sample

The HRS is a nationally representative panel study of noninstitutionalized adults older than age 50, with an oversampling of African Americans, Hispanics, and Florida residents (Heeringa & Connor, 1995). The HRS was launched in 1992, and the study participants were followed up and interviewed biennially. Individuals from a new birth cohort were added to the study every 6 years, in order to maintain a sample representative of the population older than age 50 in the United States. The HRS was approved by the University of Michigan Institutional Review Board. We analyzed six waves (Wave 8–2006 through Wave 13–2016) of the HRS. These six waves were selected for this analysis because the HRS began collecting psychosocial data, which includes measures of social support and discrimination, from respondents in 2006, and the 2016 wave is the most recent data available. Psychosocial data, also referred to as Leave-Behind (LB) data, were collected using questionnaires that were left with the participants at the end of their in-home interviews. Participants were requested to complete and mail back these surveys to the study team. Psychosocial information was collected in each biennial wave from a rotating random 50% subsample of the panel participants. In order to analyze data from the complete sample, we concatenated data from the 2006 and 2008, 2010 and 2012, and 2014 and 2016 waves to create a three-wave longitudinal data set (HRS, 2019). For the present study, the sample selection criteria were (a) self-identification as African American, (b) 65 years of age or older, and (c) nonproxy respondents. In total, 14,808 participants responded to the LB questionnaire. After excluding participants younger than 65 years of age (*N* = 5,312), we obtained a sample size of 9,496, of which 1,154 were African Americans. After excluding proxy respondents (*N* = 10), the final analytic sample size was 1,144.

### Measures

#### Outcome variable

*Depressive symptoms* were measured using the eight-item Center for Epidemiologic Studies Depression scale. Respondents were asked whether they experienced the following symptoms all or most of the time during the last week: (a) felt depressed, (b) everything is an effort, (c) sleep is restless, (d) felt alone, (e) felt sad, (f) could not get going, (g) felt happy, and (h) enjoyed life. Dichotomous responses of 1 = yes and 0 = no were used for each item (Bugliari et al., 2019). Positive valence items were reverse coded and summed scores ranged from 0 to 8, with higher scores indicating more depressive symptoms. The Cronbach’s alphas for the sample ranged from 0.77 (2016) to 0.81 (2014).

#### Predictor variables

*Everyday discrimination* was measured using a six-item scale adapted from the Everyday Discrimination Scale (Williams et al., 1997), which assessed episodes of unfair treatment experienced in day-to-day life. Response categories ranged from 1 (*almost everyday*) to 6 (*never*). We reverse coded the response categories and recoded items on a 0 (*never*) to 5 (*almost everyday*) scale. Items were averaged, with higher scores indicating more frequent experiences of everyday discrimination. The final score was set to missing if there were more than three items with missing values (Smith et al., 2017). If respondents had missing data on three or fewer items of the six-item scale, the missing items were assigned as missing and excluded in creating the final score. For example, if one respondent rated five items but missed one item, we computed the final score by summing up the five valid items and then dividing the sum by 5. The Cronbach’s alphas of this scale for the selected sample ranged from 0.79 (2006) to 0.83 (2014).

A four-item scale was utilized to measure *frequency of contact with family*. Respondents were asked: “On average, how often do you do each of the following with any of your family members, not counting any who live with you? 1) meet up (including both arranged and chance meetings), 2) speak on the phone, 3) write or email, and 4) communicate by Skype, Facebook, or other social media.” Responses were recorded on a 1 (*three or more times a week*) to 6 (*less than once a year or never*) scale. The items were reverse coded and averaged, with higher scores indicating more frequent contact with the family. The final score was set to missing if there was more than one item with missing values (Smith et al., 2017).

A three-item scale was utilized to measure *perceived social support from family*. Specifically, respondents were asked to rate how they felt about their family members relative to each of the following statements: (a) How much do they really understand the way you feel about things? (b) How much can you rely on them if you have a serious problem? (c) How much can you open up to them if you need to talk about your worries? Responses were recorded on a 1 (*a lot*) to 4 (*not at all*) scale. Scales were reverse coded and averaged, with higher scores indicating more support. The final score was set to missing if there was more than one item with missing values (Smith et al., 2017). For respondents who missed only one item of the three-item scale, the missing item was assigned as missing and excluded in creating the final score. For example, if one respondent rated two items but missed one item, we computed the final score by summing up the two valid items and then dividing the sum by 2. *Frequency of contact with friends* and *perceived social support from friends* were assessed by similar questions and response options used for the family variables.

#### Covariates

Demographic factors and socioeconomic status were controlled in the present study because of their possible associations with depressive symptoms. Specific demographic variables included *years of age*, *gender* (male/female), and *marital status*. Marital status was dichotomized into (a) married/partnered and (b) divorced, separated, widowed, or never married. The socioeconomic status indicators included *years of education* and *annual household income* (in dollars). The annual household income was positively skewed (skewness = 3.63). Due to the skewed distribution, household income was log-transformed for multivariate analyses. Overall, all variables were time-varying except gender and education.

### Analysis Strategy

Descriptive statistics were conducted at baseline to capture the sample characteristics. Mixed-effects negative binomial regression was used to estimate the influence of discrimination, frequency of contact, and social support on depressive symptoms among older African Americans. Mixed-effects negative binomial regression was used because the distribution of depressive symptoms, which was treated as a count variable in the HRS, was non-normal and overdispersed (*M* =1.61, *S*^2^ = 3.78). The interaction terms discrimination × frequency of contact and discrimination × social support were used to test the stress-buffering effects of these two social relationship variables. All multivariate analyses controlled for sociodemographic variables and accounted for the complex sample design of the HRS by applying HRS-provided survey weights specifically for LB participants (Smith et al., 2017). To ease the interpretation of interactions, we created graphs to show the predicted probability of depressive symptoms for significant interaction terms.

## Results

### Sample Characteristics

Table 1 presents unweighted and weighted sample characteristics for the study variables among older African Americans at baseline. The average age for the study sample was 73 years. The majority of respondents were women (61.1%) and those who were unmarried or unpartnered (58.7%). On average, respondents had 11 years of education and a household income of approximately $30,000. In terms of discrimination, respondents reported an average score of 0.70. In general, respondents reported similar levels of contact with friends (*M* = 3.52) and extended family (*M* = 3.45), and close to equal levels of perceived support from extended family (*M* = 3.17) and friends (*M* = 3.12). On average, respondents reported having approximately two symptoms of depression during the previous week (*M* = 1.87).

**Table 1.** Sample Characteristics at Baseline (unweighted *N* = 1,144, weighted *N* = 6,510,641)

Study variables	Unweighted	Weighted	Range
Age (in years)	73.45 (6.89)	74.37 (7.22)	65–100
Gender (%)			
Male	36.8	38.9	
Female	63.2	61.1	
Marital status (%)			
Married/partnered	43.83	41.3	
Divorced/separated/widowed/never married	56.17	58.7	
Education (in years)	11.04 (3.42)	10.63 (3.64)	0–17
Annual household income (in USD)	30,287 (33,680)	28,580 (35,660)	0–319,830
Everyday discrimination	0.67 (0.81)	0.70 (0.84)	0–5
Frequency of contact with friend	3.59 (1.04)	3.52 (1.05)	1–6
Perceived social support from friend	3.12 (0.74)	3.12 (0.75)	1–4
Frequency of contact with family	3.48 (1.08)	3.45 (1.09)	1–6
Perceived social support from family	3.18 (0.78)	3.17 (0.78)	1–4
Depressive symptoms (CES-D)	1.61 (1.94)	1.87 (2.07)	0–8

*Notes*: CES-D = Center for Epidemiologic Studies Depression scale; LB = Leave-Behind. Mean and SD, presented in parentheses, are presented for continuous variables. Percentages are presented for categorical variables. Survey weights for LB participants were applied to generate the population estimates.

### Mixed-Effects Negative Binomial Regression Results

Table 2 presents the incidence rate ratios from mixed-effects negative binomial regression analyses. The likelihood-ratio tests of alpha for all five models were statistically significant, indicating that the negative binomial models were more appropriate than the Poisson models for the study sample. Also, the likelihood-ratio tests of the fitted models versus null models (constant-only models) were significant, indicating that the fitted models had a better fit than the null models.

**Table 2.** Incidence Rate Ratios for Independent Variables Associated With Depressive Symptoms Among Older African Americans From Mixed-Effects Negative Binomial Regression

Independent variables	Model 1	Model 2	Model 3	Model 4	Model 5
Age (in years)	1.00 (0.99–1.01)	1.00 (0.99–1.01)	1.00 (0.99–1.01)	1.00 (0.99–1.01)	1.00 (0.99–1.01)
Gender					
Male	—	—	—	—	—
Female	1.28 (1.08–1.52)**	1.28 (1.07–1.52)**	1.28 (1.08–1.52)**	1.28 (1.08–1.52)**	1.27 (1.07–1.51)**
Marital status					
Married/partnered	—	—	—	—	—
Divorced/separated/widowed/never married	0.98 (0.81–1.17)	0.98 (0.81–1.18)	0.98 (0.82–1.18)	0.97 (0.81–1.17)	0.99 (0.82–1.19)
Education (in years)	0.91 (0.89–0.94)***	0.91 (0.89–0.94)***	0.91 (0.89–0.93)***	0.91 (0.89–0.93)***	0.91 (0.89–0.93)***
Annual household income (log-transformed)	0.78 (0.71–0.86)***	0.78 (0.71–0.86)***	0.78 (0.71–0.86)***	0.78 (0.71–0.86)***	0.79 (0.71–0.86)***
Everyday discrimination	1.24 (1.16–1.33)***	1.24 (0.97–1.57)	0.94 (0.73–1.20)	1.05 (0.86–1.30)	0.97 (0.77–1.24)
Frequency of contact with friends	0.98 (0.91–1.07)	0.98 (0.90–1.08)	0.99 (0.91–1.07)	0.98 (0.90–1.06)	0.98 (0.91–1.07)
Perceived social support from friend	1.00 (0.91–1.10)	1.00 (0.91–1.10)	0.94 (0.83–1.06)	1.00 (0.91–1.10)	1.00 (0.91–1.10)
Frequency of contact with family	0.96 (0.90–1.04)	0.96 (0.90–1.04)	0.96 (0.89–1.03)	0.93 (0.86–1.02)	0.96 (0.90–1.04)
Perceived social support from family	0.94 (0.86–1.03)	0.94 (0.86–1.03)	0.94 (0.86–1.03)	0.94 (0.86–1.03)	0.89 (0.80–0.99)*
Everyday discrimination × Frequency of contact with friends		1.00 (0.94–1.07)			
Everyday discrimination × Perceived social support from friends			1.09 (1.01–1.18)*		
Everyday discrimination × Frequency of contact with family				1.05 (0.99–1.11)	
Everyday discrimination × Perceived social support from family					1.08 (1.01–1.17)*
Survey wave	1.02 (0.95–1.10)	1.02 (0.95–1.10)	1.02 (0.95–1.10)	1.02 (0.95–1.10)	1.03 (0.95–1.10)
Model specifics					
LR test of α = 0: χ ^2^ (01)	703.53***	703.25***	703.20***	702.75***	703.62***
LR test of fitted vs. null model: χ ^2^ (*df*)	217.01 (11)***	217.93 (12)***	222.81 (12)***	216.19 (12)***	211.97 (12)***

*Notes*: *df* = degrees of freedom; LR test = likelihood-ratio test; Survey weights for Leave-Behind participants were applied. 95% confidence intervals in parentheses.

*p < .05, **p < .01, ***p < .001.

**Figure 1.**
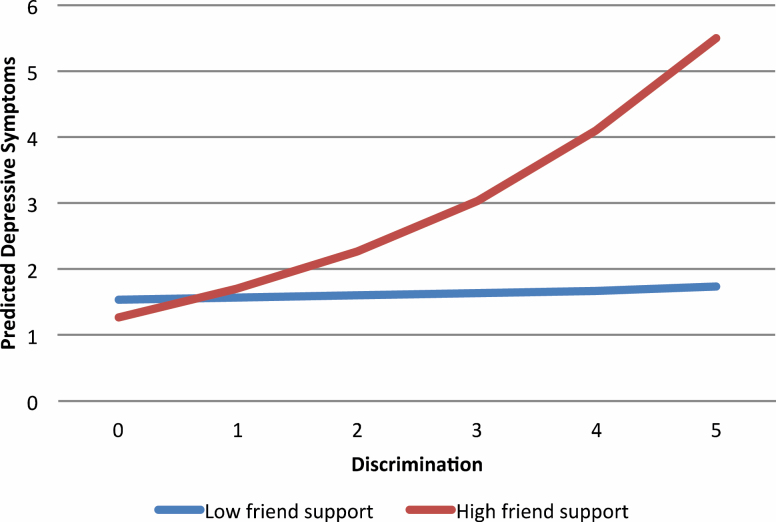
Predicted count of depressive symptoms by discrimination and social support from friends among older African Americans.

**Figure 2.**
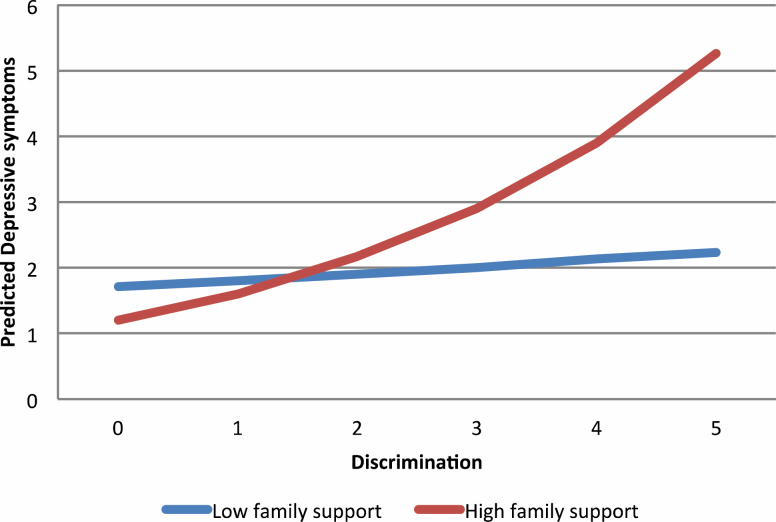
Predicted count of depressive symptoms by discrimination and social support from extended family among older African Americans.

Model 1, in which depressive symptoms were regressed on discrimination and social relationship variables, demonstrated that a one-point increase in discrimination score predicted 1.24 times the rate of depressive symptoms over time (incidence rate ratio = 1.24, 95% confidence interval = 1.16–1.33). However, contact frequency and emotional support from family and friends were statistically unrelated to depressive symptoms in older African Americans. Models 2 and 3 tested the effects of the discrimination × friend contact and discrimination × friend support interaction terms, respectively, on depressive symptoms over time. Although the interaction between contact with friends and discrimination was not significant, the interaction between friend support and discrimination achieved statistical significance. This interaction showed that discrimination and depressive symptoms were not associated over time for older African Americans who reported low levels of friend support (Figure 1). However, among older African Americans who reported high levels of support from friends, more frequent experiences of discrimination were associated with more depressive symptoms over time. Models 4 and 5 tested the effects of the discrimination × family contact and discrimination × family support interaction terms, respectively, on depressive symptoms over time. The interaction between discrimination and family contact did not achieve statistical significance, but the interaction between discrimination and family support was a significant predictor of depressive symptoms over time (Figure 2). Specifically, this interaction revealed that more frequent experiences of discrimination were associated with more depressive symptoms over time. The magnitude of this association varied by levels of family support. Among respondents who reported low levels of support from family, the strength of this association was very weak. However, the association between discrimination and depressive symptoms over time was substantially stronger among respondents who reported high levels of support from family.

## Discussion and Implications

This study is among the first to examine whether support from friends and extended family has long-term moderating effects in the relationship between discrimination and depressive symptoms among older African Americans. This study contributes to our understanding of the competing models of social support (stress-buffering resource vs. stress-coping resource) and the role of the structure (frequency of contact) and function (social support) of the social network in protecting mental health, by providing evidence that support may serve as a coping resource for older African Americans dealing with everyday discrimination. Furthermore, we extended previous research by testing longitudinal associations focusing solely on within-group differences among older African Americans. Within-group differences are important to better understand the specific moderating effects of social support purely within African Americans, which is often obscured in comparison studies. In addition, while previous research using HRS data has explored the relationship between discrimination and mental health, these studies have focused on racial differences (Ayalon & Gum, 2011). Given that everyday discrimination may be tapping into differing underlying concepts and carry nonequivalent meanings for African Americans and whites (Kim et al., 2014), focusing on discrimination solely among African Americans allows us to generate more specific and nuanced knowledge on its effects in this population.

Consistent with findings from previous research, both cross-sectional (Marshall-Fabien & Miller, 2014) and longitudinal (English et al., 2014), we found that more frequent experiences of everyday discrimination are associated with more depressive symptoms in older African Americans over time. These findings suggest that everyday discrimination can be perceived as a stressor that negatively affects mental health. Everyday discrimination taps into daily hassles and indignities that are disproportionately experienced by minority groups (e.g., racial/ethnic, sexual/gender, and religious; Cortina, 2008). These experiences constitute chronic stressors. Although experiences of everyday discrimination lack the intensity of major experiences of discrimination (e.g., being unfairly fired, being unfairly stopped and searched by the police), they are nevertheless detrimental to mental health, as the chronic and cumulative nature of everyday discrimination can wear down or “weather away” an individual both mentally and physically (Geronimus, 1992; Wheaton, 1997). Therefore, older African Americans who experience everyday discrimination, a chronic stressor, are at heightened risk of poor mental health.

Interestingly, contact frequency with family and friends did not have an independent effect on depressive symptoms among older African Americans. Extant studies on the frequency of contact and depressive symptoms are equivocal, with some studies finding no significant associations between frequency of contact with friends and family and depressive symptom (Taylor et al., 2018) while other studies found that frequency of contact protects against depressive symptoms (Nguyen et al., 2019). It is possible that the objective qualities of social networks, such as frequency of contact and network size, do not protect against depressive symptoms or are not as important as subjective and qualitative aspects of social networks and relationships in the promotion of mental health. Subjective relational measures, such as relationship satisfaction and emotional closeness to family and friends, tap into an individual’s perceptions and appraisal of their relationships, which may be more significant for stress coping and well-being (Nguyen, Chatters, Taylor, & Mouzon, 2016).

In addition, we found no independent effects for social support from friends and family in relation to depressive symptoms. This null finding may be due to the fact that older African Americans’ perceptions of the adequacy and quality of support may be more important than enacted support for mental health. Additional research is necessary to further understand the role of social support in the mental health of older African Americans, as well as the impact of perceptions of support on mental health.

The significant interactions in this analysis indicated that social support from family and friends moderated the relationship between discrimination and depressive symptoms over time. Specifically, among older African Americans with higher levels of social support from friends and family, more frequent experiences of discrimination predicted more depressive symptoms over time, whereas experiences of discrimination and depressive symptoms were either not related or weakly related among those with lower levels of social support. Instead of social support reducing or buffering against the deleterious effects of discrimination as we had hypothesized, these findings revealed that social support operated within the resource mobilization framework (Wheaton, 1985). The resource mobilization framework asserts that when stressful events are perceived as unmanageable, its negative psychological sequelae ensue. In order to ameliorate this, individuals reach out to network members to mobilize support that is necessary for coping with stressful events. Regarding the current study findings, it is possible that older African Americans who experienced everyday discrimination more frequently and increased depressive symptoms as a result more frequently sought social support from friends and extended family to cope with this. According to the resource mobilization framework, members of an individual’s network may also observe the individual’s heightened negative psychological response to the stressor and respond by mobilizing their support for the individual. Related to our findings, friends and family members may provide more support when they notice more severe signs of depression in respondents who reported more frequent experiences of discrimination. Additionally, ancillary analysis (not shown) indicated that more frequent experiences of everyday discrimination at baseline are associated with increased social support from friends at subsequent waves, which provides additional support for the resource mobilization framework (Wheaton, 1985). Altogether, this finding indicates that social support from friends and extended family serves as an important stress-coping resource for older African Americans. Evidence supporting the function of social support as a coping resource has been documented in previous studies (McFarlane et al., 1983; Nguyen, Chatters et al., 2017). The present study extends previous work by using a longitudinal study design to reveal that social support functions as a longer-term coping resource for older African Americans who experience more frequent everyday discrimination and lends evidence to the resource mobilization framework.

The present study did not identify a moderating effect for the frequency of contact on the association between discrimination and depressive symptoms. This indicates that the frequency of contact functions differently from social support in the stress-coping process. Frequency of contact is an objective measure of relationships that does not capture subjective relational characteristics, such as the quality of social interactions and the individual’s satisfaction with their interactions. As we have previously discussed, subjective characteristics may be more instrumental for mental health and the stress-coping process than objective relational characteristics.

### Implications

The present findings provide important implications for practice with and interventions for older African Americans. First, practitioners should focus their efforts on developing and tailoring interventions that help older African Americans identify and mobilize stress-coping resources in their social networks. Specifically, instead of focusing predominately on the structure of networks (i.e., network size and frequency of contact), interventions should also focus on the function and quality of older African Americans’ networks, such as relationship satisfaction, social support, and satisfaction with support. For example, interventions could focus on helping clients build and enhance their relationships with friends and extended family members, on whom they can rely for dealing with life stressors, such as everyday discrimination. Also, interventions that include network members who are important to the client, such as close friends and family members, could increase the effectiveness of mental health services for older African Americans. Moreover, network changes (e.g., death of a friend or relocation of an adult child) can affect both frequency of contact and the level of social support in older adults. Thus, practitioners should be mindful of how these network changes can affect clients’ available coping resources for dealing with life stressors. Finally, in assessing older African Americans’ social resources and level of social integration, practitioners should include assessments of network function and quality.

Future research could further test the mechanisms underlying the pathways in which social support moderates the impact of stressors on mental health. Our findings suggest that social support is mobilized around those whose mental health is most affected by discrimination. However, the precise mobilization process remains unknown. Are older African Americans more likely to reach out to their networks and seek support in the face of a stressful event, or do their network members recognize their struggles and offer increased support? It is also important to investigate factors that motivate older African Americans to seek social support, which has significant practice implications. Additionally, future research could further test whether everyday discrimination predicts an accelerated increase in depressive symptoms and provide a better understanding of the pathway linking discrimination, social support, and mental health in older African Americans.

### Limitations and Strengths

The study has several limitations. First, there are four types of social support (Thoits, 1982), namely emotional support (perceived love and empathy), instrumental support (tangible goods or services), informational support (available environmental resources), and appraisal support (self-evaluation). The measures of the social support used in the HRS data set do not assess all support types. Thus, it remains unknown as to which specific type of social support is more effective as a stress buffer or coping resources over time. Second, the present findings were based on an observational study design using self-reported measures, and the causal relationship among discrimination, social support, and depressive symptoms cannot be determined. Furthermore, self-reported measures were subject to recall and social desirability bias. Third, the inclusion of limited missing values in the creation of mean scores for everyday discrimination and perceived support may reduce these measures’ construct validity. However, this scaling method is consistent with recommendations from the investigators of the HRS, and it allowed for a larger sample size and increased statistical power with acceptable psychometric properties (Smith et al., 2017). Fourth, due to the overall low counts of depressive symptoms in the present study, the study findings may not be generalizable to older African Americans with severe depression or clinically diagnosed depressive disorders. Last, while our study sample is representative of the older African American population, our study findings have limited generalizability to groups excluded from the sample such as middle-aged adults and racial and ethnic groups other than African Americans.

It is equally important to note the strengths and contributions of this analysis. The present study contributes to the very limited literature on the longitudinal role of social support in coping with discrimination among older African Americans. Given that African Americans disproportionately experience everyday discrimination, which research has consistently linked to poor mental health (Nguyen, Taylor et al., 2017; Williams et al., 1997), it is imperative to identify social resources that help older African Americans cope with discrimination and inform practice and interventions within this population. The present study indicates that supportive friends and family members are important for coping with the chronic stressors of everyday discrimination in the long term. Another strength of this study is the use of a nationally representative sample of older African Americans. The use of nationally representative data allows for population-level inferences. In addition, we use mixed-effects models that can include both time-varying and time-invariant variables, which allow us to examine the relationships between discrimination and depressive symptoms that vary across three waves. Also, by incorporating both fixed and random effects, mixed-effects models distinguish between-subject and within-subject sources of variability (Fitzmaurice et al., 2004, p. 188), which allow us to accommodate the nonindependence of observations due to repeated measurements.

## Conclusions

Overall, we found that everyday discrimination was positively associated with depressive symptoms among older African Americans over time, and older African Americans who experience more discrimination and depressive symptoms also receive more support from their family and friends. These findings further confirm and expand the utility of the resource mobilization framework in investigations of social support as a resource for coping with day-to-day stressors. By examining social support longitudinally, we found that support from friends and extended family serves as important coping resources over time. It is noteworthy that not all relationship characteristics function equally, as subjective relational qualities (i.e., social support) play a more important role in stress coping and promotion of mental health than objective relational qualities (i.e., frequency of contact). This underscores the importance of investigating both subjective and objective qualities of family and friend relationships. Collectively, these findings advocate for more in-depth investigations of the role of extended family and friends in the mental health of older African Americans and advance the literature on discrimination and mental health in this population.
